# Supplementary material for: ProteinShader: illustrative rendering of macromolecules
Source: BMC Struct Biol. 2009 Mar 30;9:19. doi: 10.1186/1472-6807-9-19 (PMC2672931; doi:10.1186/1472-6807-9-19)
Supplement: Additional file 1 — ProteinShader program without source code. This compressed file contains the complete ProteinShader program including associated libraries, but no source code. A README.txt file gives an overview of the ProteinShader distribution, and the index.html file in the help subdirectory has directions on getting started with the program as well as a set of tutorials. [file 1472-6807-9-19-S1.zip › ProteinShader-beta-0_9_4-binary/help/api/org/proteinshader/structure/enums/class-use/AminoAcidEnum.html]

Uses of Class org.proteinshader.structure.enums.AminoAcidEnum (ProteinShader API)


|  |  |  |
| --- | --- | --- |
| **Overview** \| **Package** \| **Class** \| **Use** \| **Tree** \| **Deprecated** \| **Index** \| **Help** | |  |
| PREV   NEXT | **FRAMES**    **NO FRAMES**     **All Classes** |  |


---


## **Uses of Class org.proteinshader.structure.enums.AminoAcidEnum**

| Packages that use AminoAcidEnum | |
| --- | --- |
| **org.proteinshader.structure** | Holds the classes that store information from a Protein Data Bank file: Structure, Model, Chain, AminoAcid, Heterogen, Water, Atom, Bond, Helix, BetaStrand, Loop, *etc*. |
| **org.proteinshader.structure.enums** | Holds the enums needed by org.proteinshader.structure and its subpackages. |
| **org.proteinshader.structure.visitor** | Holds Visitor classes that know how to traverse the hierarchy of objects held by class Structure. |

Uses of AminoAcidEnum in org.proteinshader.structure

| Methods in org.proteinshader.structure that return AminoAcidEnum | |
| --- | --- |
| `AminoAcidEnum` | `Segment.getAminoAcidType()`             Returns the type of AminoAcid that this Sement represents. |
| `AminoAcidEnum` | `Atom.getAminoAcidType()`             If the Atom belongs to an AminoAcid, this method will return the AminoAcid type. |
| `AminoAcidEnum` | `AminoAcid.getType()`             Returns the type of amino aicd: ALA, ARG, ASN, *etc.* The type cannot be null because it is checked by the constructor. |

| Methods in org.proteinshader.structure with parameters of type AminoAcidEnum | |
| --- | --- |
| `AminoAcid` | `Chain.addNewAminoAcid(AminoAcidEnum type, String residueID)`             Creates a new AminoAcid with the type and Residue ID given as arguments, adds the AminoAcid to the Chain's collection of AminoAcids, and returns a reference to the new AminoAcid. |
| `void` | `Atom.setAminoAcidType(AminoAcidEnum aminoAcidType)`             Marks the Atom as belonging to a particular type of AminoAcid. |

Constructors in org.proteinshader.structure with parameters of type AminoAcidEnum

`AminoAcid(AminoAcidEnum type,
String residueID,
String chainID,
String modelID,
String structureID)`
  
          Constructs an AminoAcid with the type and IDs given as arguments.

Uses of AminoAcidEnum in org.proteinshader.structure.enums

| Methods in org.proteinshader.structure.enums that return AminoAcidEnum | |
| --- | --- |
| `static AminoAcidEnum` | `AminoAcidEnum.valueOf(String name)`             Returns the enum constant of this type with the specified name. |
| `static AminoAcidEnum[]` | `AminoAcidEnum.values()`             Returns an array containing the constants of this enum type, in the order they're declared. |

Uses of AminoAcidEnum in org.proteinshader.structure.visitor

| Methods in org.proteinshader.structure.visitor with parameters of type AminoAcidEnum | |
| --- | --- |
| `boolean` | `BondPredictor.isDoubleBond(Atom srcAtom, Atom dstAtom, AminoAcidEnum aminoAcidType)`             Uses the Atom IDs (from Atom name field in a PDB record) and the AminoAcid type to determine if the Atoms should have a double Bond (assuming that they both belong to the same AminoAcid). |
| `void` | `Visitor.setMode(AminoAcidEnum aminoAcidType)`             Restricts the traversal to a particular AminoAcid type (this mode can also be used to restrict which Segments are visited). |

---


|  |  |  |
| --- | --- | --- |
| **Overview** \| **Package** \| **Class** \| **Use** \| **Tree** \| **Deprecated** \| **Index** \| **Help** | |  |
| PREV   NEXT | **FRAMES**    **NO FRAMES**     **All Classes** |  |


---

# *Copyright © 2007-2008*
